# Uncomplicated falciparum malaria among schoolchildren in Bajil district of Hodeidah governorate, west of Yemen: association with anaemia and underweight

**DOI:** 10.1186/s12936-020-03431-1

**Published:** 2020-10-07

**Authors:** Talal S. Alwajeeh, Rashad Abdul-Ghani, Amal F. Allam, Hoda F. Farag, Safia S. M. Khalil, Amel Y. Shehab, Mona H. El-Sayad, Raed A. Alharbi, Shaia S. R. Almalki, Ahmed A. Azazy

**Affiliations:** 1 Laboratory Department, Kuwait University Hospital, Sana’a University, Sana’a, Yemen; 2 Department of Medical Parasitology, Faculty of Medicine and Health Sciences, Sana’a University, Sana’a, Yemen; 3 Tropical Disease Research Center, Faculty of Medicine and Health Sciences, University of Science and Technology, Sana’a, Yemen; 4 Department of Parasitology, Medical Research Institute, Alexandria University, Alexandria, Egypt; 5 Laboratory Medicine Department, Faculty of Applied Medical Sciences, Al Baha University, Al Baha, Saudi Arabia

**Keywords:** Falciparum malaria, Malnutrition, Haematological indices, Schoolchildren, Yemen

## Abstract

**Background:**

Malaria, malnutrition and anaemia are major public health problems in Yemen, with Hodeidah being the most malaria-afflicted governorate. To address the lack of relevant studies, this study was conducted to determine the prevalence of *Plasmodium falciparum* and its relation to nutritional status and haematological indices among schoolchildren in Bajil district of Hodeidah governorate, west of Yemen.

**Methods:**

A cross-sectional study was conducted among 400 schoolchildren selected randomly from four schools in Bajil district. Data about demographic characteristics, risk factors and anthropometric measurements of age, height and weight were collected. Duplicate thick and thin blood films were prepared, stained with Giemsa and examined microscopically for malaria parasites. The density of *P. falciparum* asexual stages was estimated on thick films. EDTA-blood samples were examined for the haematological indices of haemoglobin (Hb) and blood cell counts.

**Results:**

*Plasmodium falciparum* was prevalent among 8.0% (32/400) of schoolchildren with a mean parasite density of 244.3 ± 299.3/µL of blood and most infections showing low-level parasitaemia, whereas *Plasmodium vivax* was detected in one child (0.25%). Residing near water collections was a significant independent predictor of falciparum malaria [adjusted odds ratio (AOR) = 2.6, 95.0% CI 1.20–5.72; *p* = 0.016] in schoolchildren. Mild anaemia was prevalent among more than half of *P. falciparum*-infected schoolchildren and significantly associated with falciparum malaria (AOR = 5.8, 95.0% CI 2.39–14.17; *p* < 0.001), with a mean Hb concentration of 10.7 ± 1.0 g/dL. Although the mean values of the total white blood cells, monocytes and platelets were significantly lower in infected than non-infected schoolchildren, they were within normal ranges. More than half of the children were malnourished, with stunting (39.3%) and underweight (36.0%) being the most prevalent forms of malnutrition; 6.3% of children were wasted. Underweight (AOR = 5.3, 95.0% CI 2.09–13.62; *p* < 0.001) but not stunting or wasting, was a significant predictor of falciparum malaria among schoolchildren.

**Conclusion:**

Asymptomatic falciparum malaria is prevalent among schoolchildren in Bajil district of Hodeidah Governorate, with predominance of low parasitaemic infections and significant association with mild anaemia and underweight. Residence near water collection is a significant predictor of infection with falciparum malaria among schoolchildren. Further studies among children with severe malaria and those with high parasite densities are recommended.

## Background

Malaria represents a major health challenge in tropical and sub-tropical countries, including Yemen. In 2018, 228 million malaria cases and 405,000 malaria-related deaths have been estimated worldwide [1]. In Yemen, approximately two-thirds of the population are at risk of infection, with over 117,000 confirmed cases and 57 deaths being reported in 2018 [1]. Malaria in Yemen is predominantly caused by *Plasmodium falciparum* [1]. Hodeidah, a coastal governorate in Tihama Region, west of Yemen, is the most afflicted governorate with malaria [2, 3]. Although substantial reductions in malaria burden have been achieved since the launch of the National Malaria Control Programme (NMCP) in 2000, malaria control became challenging because of the conflict repercussions, such as people displacement and humanitarian crisis.

Child malnutrition is high in developing countries, particularly among rural residents [4]. In Yemen, the ongoing war and poverty exacerbate the humanitarian crisis and food insecurity, leading to high rates of acute and severe acute malnutrition (SAM) among children in active-conflict and access-restricted governorates such as Hodeidah [5]. Anthropometry is a useful tool to assess the growth and nutritional status of children through physical measurement of weight, height and body mass index [6, 7]. Nutritional status can be assessed and classified using anthropometric indices called Z scores or standard deviation (SD) units [7]. These are calculated based on age, height and weight measurements and include height-for-age (HAZ), weight-for-age (WAZ) and weight-for-height (WHZ) [7], where Z-score cut-off values of 2 SD units below the reference medians are recommended to classify low anthropometric levels. Low HAZ, WHZ and WAZ scores indicate stunting, wasting and underweight, respectively [6].

Nutritional status could be a critical modulator of malaria morbidity and mortality [8]. A recent systematic review revealed the complexity and controversy in the interactions between malaria and nutritional status [9]. Although malnutrition can increase the risk of malaria and worsen malaria morbidity and mortality [10–13], it can confer some protection against symptomatic malaria [14–16]. Malaria can also be a risk factor for malnutrition [17]. Early diagnosis and treatment are unlikely in children suffering from SAM because they rarely exhibit clinical manifestations such as fever, making them at the highest risk of severe disease and death [18]. Therefore, proactive screening of children with SAM in endemic areas has been suggested irrespective of the presence of symptoms.

Falciparum malaria can lead to changes in the haematological indices of infected individuals, including those related to the major cell lines of red blood cells (RBCs), white blood cells (WBCs) and platelets [19–21]. These changes should be considered to improve diagnosis and treatment and predict malaria consequences in different epidemiologic settings. Falciparum malaria could lead to haematological abnormalities, such as anaemia and thrombocytopaenia, which can play a role in the pathogenesis and complications of the disease [21]. These changes during falciparum malaria among Yemeni children are yet to be elucidated. Therefore, this study aimed to determine the prevalence of uncomplicated falciparum malaria in relation to haematological and nutritional indices among schoolchildren in Bajil district of Hodeidah governorate, west of Yemen.

## Methods

### Study design, setting and population

**Fig. 1:**
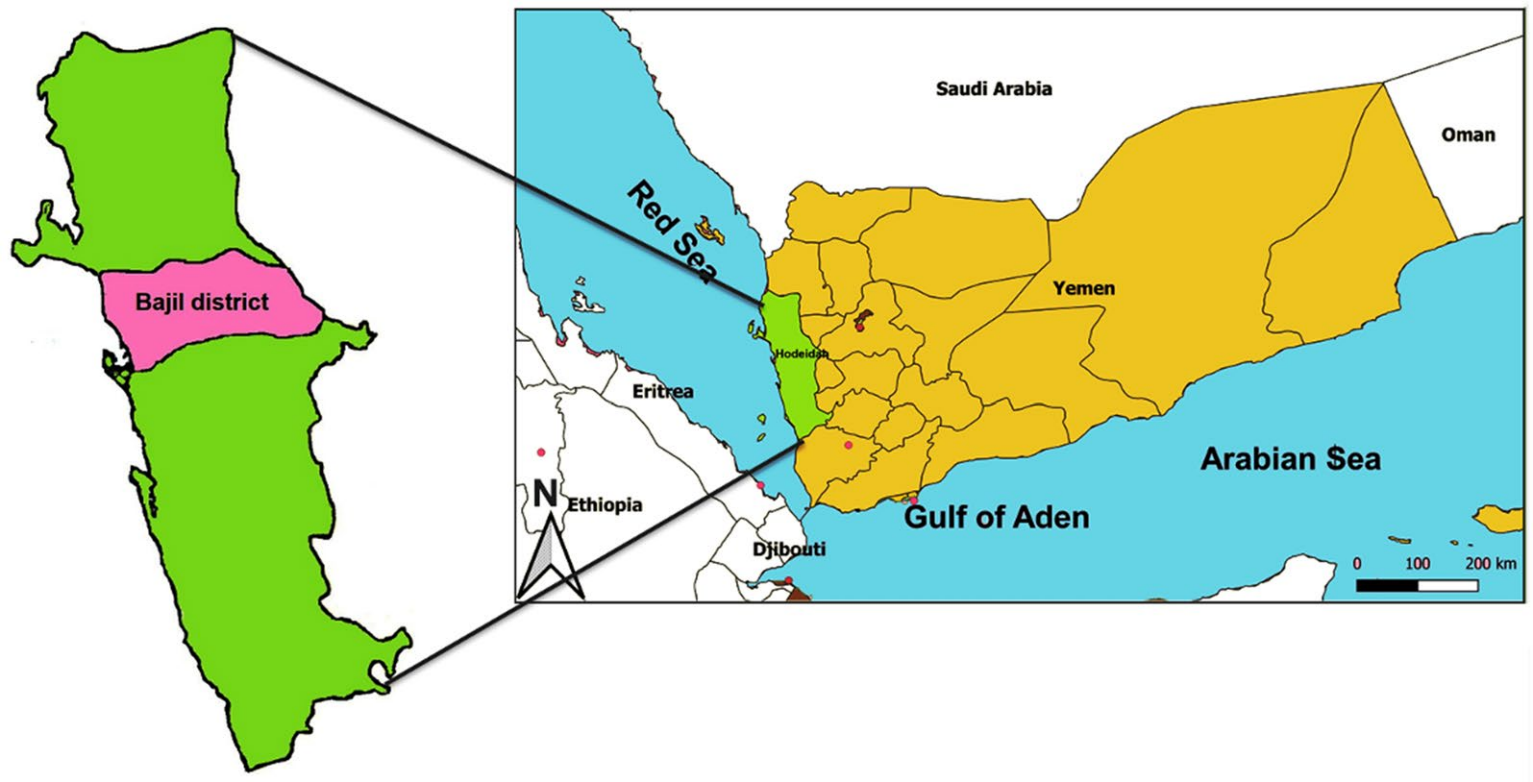
Map of Yemen showing the locations of Hodeidah governorate and Bajil district

A school-based, cross-sectional study was conducted in Bajil district of Hodeidah during the malaria transmission season (November 2017 to January 2018). Malaria Unit of the NMCP in the district is one of the sentinel sites for monitoring of anti-malarial drug efficacy and resistance. Bajil district is located at the geographic coordinates of 15° 06′ N and 43° 28′ E (Fig. 1) and inhabited by 169,884 people according to the latest census [22]. Hodeidah is characterized by two malaria transmission seasons: November to April and May to September.

Schoolchildren of both genders and aged between 7 and 15 years were included in the present study. Children reporting intake of vitamin or nutritional supplements or anti-malarial drugs over the 6 months before the survey and those who refused to give informed consent were excluded.

### Sample size and sampling strategy

A minimum sample size of 384 schoolchildren was calculated based on an expected malaria prevalence of 50.0% (due to the absence of studies among this population sub-category) and at a confidence level of 95.0% and an accepted marginal error of 5.0%. However, 400 schoolchildren were included in this study. The sampling strategy adopted a two-stage sampling approach. In the first stage, cluster sampling was used, where 110 schools in Bajil were considered as the clusters of the study. These schools were listed and categorized as rural and urban, and then four schools were randomly selected. In the second stage, simple random sampling was adopted to select schoolchildren from the records of each school, with replacement of each ineligible or absent or unwilling to participate with the next one in the school record.

### Data collection

Data about demographic characteristics (gender, age, residence) and risk factors possibly associated with malaria among schoolchildren were collected using a structured questionnaire. In addition, the axillary temperature was measured using a thermometer, and children were considered febrile if having an axillary temperature of ≥ 37.5 °C.

To assess the anthropometric measurements of schoolchildren, height was measured to the nearest 0.1 cm using a height-measuring tape and weight was measured to the nearest 0.1 kg using Omron HN286 Digital Personal Scale (Omron Healthcare, Hoofddorp, The Netherlands). Age was retrieved from birth certificates or school records. Scores of height, weight and age were then recorded in the specified fields of the questionnaire.

### Blood sample collection

Capillary blood was collected onto clean glass slides by finger-prick to make duplicate thick and thin blood films. About 3 mL of venous blood were also collected into pre-labelled EDTA blood collection tubes (Henso Medical, Hangzhou, China) under aseptic conditions for haematological investigations.

### Laboratory investigations

#### Malaria microscopy and parasite density estimation

Thick and thin blood films prepared from capillary blood samples were stained with Giemsa and examined for malaria parasites using light microscopy according to standard guidelines [23, 24] at the Parasitology Laboratory of the General Military Hospital at Hodeidah city. Blood films were considered negative if no asexual parasites had been detected after examining 100 fields. Parasite density per µL of blood was estimated by counting the asexual stages against 200 WBCs on thick films according to standard procedures and calculations [23, 24].

#### Haematological measurements

Venous blood samples were examined for complete blood counts using Sysmex XP-300™ Automated Haematology Analyser (Sysmex Corporation, Kobe, Japan) at the Haematology Laboratory of the Military Hospital at Hodeidah city. Patterns of Hb concentration (g/dL), RBC count, total and differential WBC counts and platelet count were determined. Anaemia in children was defined as Hb < 11.5 g/dL [25], while thrombocytopaenia was defined as a platelet count < 150.0 × 10^9^/L [26].

#### Statistical analysis and calculations

Data were analysed using IBM SPSS Statistics, version 21.0 (IBM Corp., Armonk, NY, USA). Frequencies and proportions were used to express categorical variables and their associations or differences were tested using Pearson’s Chi-square or Fisher’s exact tests, whichever suitable. Mean ± SD and median ± interquartile range (IQR) were used to express the continuous variables with normally and non-normally distributed data, respectively. Differences between continuous variables were tested using independent Student’s *t*-test or Mann–Whitney U test for normally and non-normally distributed data, respectively. A binary logistic regression model was developed to identify the risk factors or predictors of falciparum malaria among schoolchildren, where odds ratios (ORs) and their corresponding 95.0% confidence intervals (CIs) were reported. This was followed by multivariable logistic regression of variables to identify the independent predictors of falciparum malaria among schoolchildren, reporting the adjusted ORs (AORs) and their corresponding 95.0% CI. Differences and associations were considered statistically significant at a *p*-value of < 0.05. In the present study, fever and thrombocytopaenia were excluded from the statistical analysis of association with falciparum malaria because only two out of five febrile children were infected and only one child was thrombocytopaenic (platelet count = 95 × 10^9^/L) to avoid bias introduced by the low statistical power.

Anthropometric indices derived from height, weight and age measurements were calculated and analysed using EPINUT (EpiInfo™ 6.04, Centers for Disease Prevention and Control, Atlanta, Georgia, USA). These were expressed as SD units or Z-scores, which were then interpreted and compared according to the growth reference curves of the National Center for Health Statistics/Centers for Disease [27]. Children with HAZ and WHZ scores ≤ −2 SD were categorized as stunted (chronically malnourished) and wasted (acutely malnourished), respectively. However, those with WAZ score  ≤ −2 SD were categorized as underweight. Children who fit into one or more of these categories were considered malnourished.

## Results

### Characteristics of the study population

Table 1 shows that the medians of age and axillary temperature were 12.0 ± 2.0 years and 36.4 ± 0.60 °C, respectively. The majority of schoolchildren were males (57.8%), aged 10 years or older (80.5%), non-febrile (98.7%), non-anaemic (79.2%), and malnourished (52.0%).

**Table 1:** Characteristics of schoolchildren in Bajil district, Hodeidah governorate, Yemen enrolled in the study (2017–2018)

Characteristics	*n* (%)
Gender	
Male	231 (57.8)
Female	169 (42.3)
Age (years)	
< 10	78 (19.5)
≥ 10	322 (80.5)
Median ± IQR: 12.0 ± 2.0	
Residence	
Rural	200 (50)
Urban	200 (50)
Axillary temperature (°C)	
Median ± IQR: 36.4 ± 0.60	
Febrility status^a^	
Febrile	5 (1.3)
Non-febrile	395 (98.7)
Anaemia status^b^	
Anaemic	83 (20.8)
Non-anaemic	317 (79.2)
Nutritional status	
Normal	192 (48)
Malnourished^c^	208 (52.0)
Stunted	157 (39.3)
Wasted	25 (6.3)
Underweight	144 (36.0)

Malaria prevalence among schoolchildren in Bajil

The total number of schoolchildren enrolled in the study was 400

*IQR* interquartile range

^a^A child was considered febrile if axillary temperature was ≥ 37.5 °C

^b^A child was considered anaemic if Hb concentration was < 11.5 g/dL

^c^A child was considered malnourished if having one or more of the nutritional abnormalities (stunting, wasting and underweight)

Of 400 schoolchildren, 32 (8.0%) and one child (0.25%) were positive for *P. falciparum* and *Plasmodium vivax*, respectively. The mean density of *P. falciparum* was 244.3 ± 299.3 parasites/µL of blood, where most infections (96.9%) showed low-level parasitaemia with absence of high-level parasitaemia. Gametocytes were detected in 12 cases (37.5%) (Table 2).

**Table 2:** Parasite density and gametocytaemia among *Plasmodium falciparum*-infected schoolchildren in Bajil district, Hodeidah governorate, Yemen (2017–2018)

Variable	*n* (%)
Parasite density (parasites/μL)	
Mean ± SD: 244.3 ± 299.3	
Range: 32–1098	
Low (< 1000)	31 (96.9)
Moderate (1000–9999)	1 (3.1)
High (≥ 10,000)	0 (0.0)
Gametocytaemia	
Yes	12 (37.5)
No	20 (62.5)

The total number of *P. falciparum*-infected children was 32

*SD* standard deviation

### Sociodemographic and risk factors associated with falciparum malaria

Residence in proximity to water collections was the only factor significantly associated with increased risk of falciparum malaria among schoolchildren in bivariate analysis (OR = 2.4, 95% CI 1.14–5.05), which was also identified it as an independent predictor of malaria (AOR = 2.6, 95.0% CI 1.20–5.72) using multivariable logistic regression (Table 3).

**Table 3:** Sociodemographic and risk factors associated with falciparum malaria among schoolchildren in Bajil district, Hodeidah governorate, Yemen (2017–2018)

Variable	*N*	Falciparum malaria positivity		
		*n* (%)	OR (95% CI)	*p*-value
Gender				
Male	231	19 (8.2)	1.1 (0.52–2.24)	0.846
Female	169	13 (7.7)	Reference	
Age (years)				
< 10	78	7 (9.0)	1.2 (0.49–2.82)	0.724
≥ 10	322	25 (7.8)	Reference	
Residence				
Rural	200	17 (8.5)	1.1 (0.56–2.36)	0.713
Urban	200	15 (7.5)	Reference	
Sleeping under a mosquito net during the night preceding the survey				
Yes	137	10 (7.3)	Reference	0.710
No	263	22 (8.4)	0.9 (0.40–1.89)	
Indoor residual spraying in the past 6 months				
Yes	180	15 (8.3)	Reference	0.824
No	220	17 (7.7)	1.1 (0.53–2.24)	
Wire screening of windows				
Yes	82	3 (3.7)	Reference	0.105
No	318	29 (9.1)	0.4 (0.12–1.28)	
Residence in proximity to water collections^a^				
Yes	171	20 (11.7)	2.4 (1.14–5.05)	0.022
No	229	12 (5.2)	Reference	

*N* number examined; *n* number positive; *OR* odds ratio; *CI* confidence interval

^a^An independent predictor of falciparum malaria among schoolchildren using multivariable logistic regression analysis (AOR = 2.6, 95.0% CI 1.20–5.72; *p* = 0.016)

### Haematological indices in *Plasmodium falciparum*-infected and malaria-negative schoolchildren

The mean values of Hb concentration as well as RBC, total WBC, monocyte and platelet counts were significantly lower in *P. falciparum*-infected than malaria-negative schoolchildren. In contrast, no statistically significant difference was found between the two groups regarding the mean percentages of neutrophils, lymphocytes and eosinophils (Table 4).

**Table 4:** Comparison of haematological indices between *Plasmodium falciparum*-infected and malaria-negative schoolchildren in Bajil district, Hodeidah governorate, Yemen (2017–2018)

Indices	*P. falciparum*-infected schoolchildren (*n* = 32)	Malaria-negative schoolchildren (*n* = 368)	*p*-value
	Mean ± SD		
Hb concentration (g/dL)	10.7 ± 1.0	11.7 ± 1.1	< 0.001
RBC count (× 10^12^/L)	4.6 ± 0.5	4.9 ± 0.4	0.002
Total WBC count(× 10^9^/L)	5.5 ± 1.2	6.6 ± 1.9	0.001
Neutrophil count (%)	36.8 ± 11.8	39.5 ± 9.7	0.144
Lymphocyte count (%)	48.1 ± 11.3	47.0 ± 9.4	0.505
Monocyte count (%)	8.6 ± 2.3	7.7 ± 2.2	0.031
Eosinophil count (%)	6.3 ± 1.6	5.7 ± 1.7	0.077
Platelet count (× 10^9^/L)	297.5 ± 65.9	347.6 ± 91.0	0.002

*Hb* haemoglobin; *RBC* red blood cell; *WBC* white blood cell; *SD* standard deviation

### Association of fever and haematological and nutritional abnormalities with falciparum malaria

Anaemia (OR = 5.2, 95% CI 2.47–10.91; *p* < 0.001) was the haematological abnormality significantly associated with falciparum malaria among schoolchildren, where it was prevalent among 20.8% (83/400) of schoolchildren and 53.1% (17/32) of *P. falciparum*-infected ones. On the other hand, underweight (OR = 3.3, 95% CI 1.55–6.93), but neither stunting nor wasting, was significantly associated with falciparum malaria among the schoolchildren. Anaemia (AOR = 5.8, 95.0% CI 2.39–14.17) and underweight (AOR = 5.3, 95.0% CI 2.09–13.62) were also identified as independent predictors of falciparum malaria using multivariable logistic regression analysis (Table 5).

**Table 5:** Association of anaemia and nutritional abnormalities with falciparum malaria among schoolchildren in Bajil district, Hodeidah governorate, Yemen (2017–2018)

Abnormality	*N*	Falciparum malaria positivity		*p*-value
		*n* (%)	OR (95% CI)	
Anaemia*				
Anaemic^a^	83	17 (20.5)	5.2 (2.47–10.91)	< 0.001*
Non-anaemic	317	15 (4.7)	Reference	
Stunting				
Yes	157	12 (7.6)	0.9 (0.44–1.95)	0.833
No	243	20 (8.2)	Reference	
Wasting				
Yes	25	3 (12)	1.7 (0.46–5.76)	0.447
No	375	29 (7.7)	Reference	
Underweight*				
Yes	144	20 (13.9)	3.3 (1.55–6.93)	0.002
No	256	12 (4.7)	Reference	

*N* total examined; *n* number infected

^*^Independent predictors of falciparum malaria using multivariable logistic regression (AOR = 5.8, 95.0% CI 2.39–14.17; *p* < 0.001 for anaemia and AOR = 5.3, 95.0% CI 2.09–13.62; *p* < 0.001 for underweight)

^a^A child was identified as anaemic if Hb concentration was < 11.5 g/dL

## Discussion

No published studies have been encountered on falciparum malaria among schoolchildren from Hodeidah governorate in relation to haematological and nutritional indices. This study revealed that 8.0% of almost asymptomatic schoolchildren in Bajil district were infected with *P. falciparum* and over 96.0% of infections having low parasite densities. This prevalence is lower than the rates reported from Hodeidah based on community surveys (16.2%) in 2003 [28] and among febrile patients (15.8%) in 2009 [29]. It is lower than the rates reported among febrile children seeking healthcare in Sana’a city between 1998 and 2000 (17.3%; 130/753) and children from Taiz governorate (18.6%; 83/447) in 2006 [30, 31]. It is lower than the household-based malaria prevalence of 18.8% (136/735) reported from the southeastern governorate of Hadhramout [32], where over 99.0% of infections being caused by *P. falciparum*. However, it is substantially lower than those reported for schoolchildren from several African countries, including Malawi (60.0%), Kenya (42.0%), Uganda (27.6%), Cameroon (22.8–33.8%) and Tanzania (21.6–38.1%; 93/244) [33–39]. In contrast, it is higher than the rates reported among Kenyan children (4.3%) in a nationwide survey and asymptomatic schoolchildren from northwest Ethiopia (6.8%; 26/385) [40, 41]. The low prevalence of *P. vivax* (0.25%) among schoolchildren in the present study is in line with the low proportion of vivax malaria in Yemen [1, 32].

In Yemen, the prevalence of falciparum malaria has been reduced following the escalated control interventions of the NMCP since its launch in 2000; however, its burden among asymptomatic schoolchildren is high and can pose a threat to malaria control efforts. The asymptomatic nature of most infections in the present study could be attributed to the low-to-moderate parasitaemia levels and the absence of high-level parasitaemia. Asymptomatic cases usually go undiagnosed and untreated, potentially contributing to parasite transmission and the emergence and spread of drug resistance [42]. Gametocyte carriage by more than one-third of asymptomatic children with uncomplicated falciparum malaria in the present study poses a threat to malaria control and elimination efforts. Such a large reservoir of asymptomatic gametocyte carriers contributes considerably to potential human-to-mosquito transmission [43]. Therefore, identification and treatment of asymptomatic gametocyte carriers should be considered when tailoring malaria elimination strategies. This can be of public health significance in the context of the ongoing conflicts in the country, where the massive internal displacement of asymptomatic carriers from malaria-endemic to malaria-free areas can make the hosting communities prone to malaria epidemics. Apart from its impact on disease transmission, asymptomatic malaria can have consequences on the health and educational performance of children [34, 44, 45]. In Yemen, for example, an earlier study revealed that asymptomatic parasitaemia of *P. falciparum* can impair the cognitive function of Yemeni children [44]. The findings of the present study underscore the need for complementing the household surveys conducted by the NMCP with school-based malaria surveys to help assess the impact of control interventions. School-age children are more preferred to adults for estimating parasite prevalence and density [46, 47], and school-based malaria surveys are reliable in estimating malaria epidemiology and assessing control interventions [48, 49].

The gender, age and residence of schoolchildren were not significant predictors of falciparum malaria in the present study. The lack of significant association between age and malaria is consistent with that found among Tanzanian schoolchildren in a nationwide survey [39]. In contrast, male gender and age younger than 10 years were significantly associated with asymptomatic malaria among Ugandan and Kenyan schoolchildren [34, 38]. However, younger age was a significant predictor of malaria among primary schoolchildren from southern Malawi [36]. Differences in age association with malaria among children could be attributed, among other factors, to differences in transmission intensity [47, 50], which is lower in Yemen compared to sub-Saharan African countries.

Independent of other factors, residence near water collections was significantly associated with a 2.6-fold higher risk of falciparum malaria among schoolchildren in Bajil. Similarly, people living near water collections in Hadhramout were at significantly higher risk of malaria [32]. In the present study, approximately one-third of schoolchildren reported sleeping under mosquito nets during the malaria transmission season, with no significant association with reduced malaria prevalence. This finding is consistent with those reported for Ugandan and Cameroonian children [34, 51] but inconsistent with that reported among Malawian schoolchildren [36]. The low utilization of mosquito nets among schoolchildren is in agreement with that (19.0%) reported at the community level in Hodeidah in 2016 [52]. Low mosquito-net utilization by children, despite ownership, is common in endemic countries. For instance, the utilization of mosquito nets was reported among 19.0 and 32.4% of Kenyan and Malawian schoolchildren, respectively [36, 40]. Consequently, efforts should be made to identify the reasons for not utilizing mosquito nets by schoolchildren to tailor appropriate educational interventions.

Malnutrition represents a major public health problem in countries endemic for malaria. In Yemen, the ongoing complex emergency and humanitarian crisis besides the absence of school feeding programmes aggravate this problem among schoolchildren. The present study revealed malnutrition in more than half of schoolchildren, with stunting and wasting being the most and least prevalent forms of malnutrition, respectively. This shows that chronic malnutrition is more prevalent than acute malnutrition among schoolchildren in the study area. Such predominance of stunting is consistent with those reported from Malawi, Laos and Cameroon [36, 37, 53] but inconsistent with that reported from Mount Cameroon [51]. Of malnutrition forms, underweight was the independent predictor significantly associated with falciparum malaria among schoolchildren in the present study, where underweight children were approximately five times more likely to have falciparum malaria compared to their counterparts. This finding is in agreement with that reported among schoolchildren from high transmission settings of Uganda [32]. In contrast, stunting was the significant predictor of falciparum malaria among children from Kenya, Malawi and Laos [36, 38, 53, 54]. Both stunting and wasting were significant predictors of malaria among children from southwest Cameroon [37]. Although the specific interaction between falciparum malaria and underweight remains not fully understood, acute weight loss could be one of the nutritional consequences of falciparum malaria [12]. Therefore, longitudinal studies are needed to assess the relationship between malaria and malnutrition and the impact of malnutrition on the treatment outcome with artemisinin-based combination therapy (ACT) among schoolchildren in Hodeidah. It is noteworthy that the risk of treatment failure with ACT can increase among malnourished children [55].

Regarding the haematological indices and according to WHO’s criteria for the classification of malaria [25], mild anaemia was prevalent among more than half of *P. falciparum*-infected schoolchildren in the present study. It was an independent predictor of falciparum malaria, where anaemic schoolchildren were at approximately six times more likely to have malaria compared to non-anaemic ones. Such a significant association agrees with that among children from Kenya, Malawi and Laos [21, 36, 53], but disagrees with that among Ugandan schoolchildren [34]. However, the establishment of a causal relationship is rather difficult in such a cross-sectional study, where malaria-associated anaemia is multifactorial. These factors include, among others, mechanical or autoimmune haemolysis, splenic sequestration of infected and non-infected RBCs and suppressed erythropoiesis [56–59].

The mean values of total WBCs and monocytes were significantly lower in infected than non-infected schoolchildren, but within the normal range for both groups. Consequently, it is difficult to establish any clinical implication from such differences in schoolchildren with uncomplicated malaria. Nonetheless, haematological changes are usually common in complicated or severe malaria. Low-to-normal WBC counts are usually observed in malaria patients, mainly due to their localization outside the peripheral circulation rather than actual depletion [60]. In contrast, leukopenia was found to be significantly higher in schoolchildren with falciparum malaria compared to malaria-negative ones in Cameroon [33]. On the other hand, monocytosis could be one of the frequent haematological changes and the most important leukocytic change characterizing malaria [21].

Thrombocytopaenia can occur in *P. falciparum*-infected patients regardless of the exposure frequency or severity of the disease [61], which could be due to splenic sequestration, immune-mediated destruction and coagulation disturbances. Given that only one thrombocytopaenic child was found in the present study, the association between thrombocytopaenia and falciparum malaria was not tested statistically. Moreover, the significantly lower mean platelet count in infected than non-infected schoolchildren has no clinical implications because both were within the normal range. This could be attributed to the low-to-moderate levels of parasitaemia in uncomplicated malaria cases. In another context, thrombocytopaenia was significantly associated with falciparum malaria in Hajjah governorate, northwest of Yemen [62]. A significant association was also reported among Kenyan and Nigerian children [21, 63].

Overall, haematological indices are difficult to use in the prediction of malaria in the study district, even with significant differences between infected and non-infected schoolchildren. In this respect, mild anaemia was the only haematological abnormality observed. This could be partially attributed to the low transmission intensity in the study district compared to the studies in African countries. The impact of transmission intensity on the differences in haematological indices could not be ruled out [64]. The utility of haematological indices as indicators of falciparum malaria should, however, be assessed in symptomatic and complicated infections and those with high parasite densities.

The present study is limited by its cross-sectional design that could not establish a causal relationship between malaria and haematological or nutritional abnormalities. Besides, its findings may not be generalizable to school-age children not enrolled in schools, who may represent a large proportion because of the ongoing complex emergency and humanitarian crisis. Nevertheless, this is the first study to provide essential information about falciparum malaria among schoolchildren in relation to haematological and nutritional indices in one of the largest districts of the most malaria-afflicted governorates in the country. Another limitation is the use of light microscopy for diagnosing malaria among schoolchildren, which overlooks sub-microscopic infections and may underestimate malaria burden in the study setting. Therefore, there is a need for the molecular-based assessment of sub-microscopic reservoir of falciparum malaria, preferably through school-based surveys, in pursuit of the efforts towards malaria elimination. Because the association between severe malaria and haematological and nutritional abnormalities could not be assessed in this school-based study, hospital-based studies are rather needed for this purpose. Longitudinal studies on the development of clinical malaria among schoolchildren with haematological and nutritional abnormalities compared to their normal counterparts are recommended.

## Conclusion

Uncomplicated falciparum malaria is prevalent among 8.0% of schoolchildren in Bajil district of Hodeidah with most infections showing parasite densities lower than 1000 parasites/µL of blood. Residence in proximity to water collections is the only risk factor significantly associated with infection, whereas demographic factors and non-use of vector control tools are not significant predictors of infection among schoolchildren. Mild anaemia is prevalent among half of *P. falciparum*-infected schoolchildren and is an independent predictor significantly associated with falciparum malaria. Other haematological indices were within the normal range in infected and non-infected schoolchildren and could not be used to predict falciparum malaria. On the other hand, more than half of schoolchildren are malnourished, predominantly being stunted and underweight. However, only underweight is a significant predictor of falciparum malaria. Further studies among children with severe malaria and high parasite densities are recommended.

## Data Availability

Data and materials are available when requested by email.

## Abbreviations

CI: Confidence interval
EDTA: Ethylenediaminetetraacetic acid
HAZ: Height-for-age Z-score
Hb: Haemoglobin
IQR: Interquartile range
NMCP: National Malaria Control Programme
OR: Odds ratio
RBC: Red blood cell
SAM: Severe acute malnutrition
SD: Standard deviation
SPSS: Statistical Packages for Social Sciences
WAZ: Weight-for-age Z-score
WBC: White blood cell
WHO: World Health Organization
WHZ: Weight-for-height Z-score
